# Supplementary material for: Using OPMs to measure neural activity in standing, mobile participants
Source: Neuroimage. 2021 Dec 1;244:118604. doi: 10.1016/j.neuroimage.2021.118604 (PMC8591613; doi:10.1016/j.neuroimage.2021.118604)
Supplement: Supplementary file 2 [file mmc2.docx]

**Supplementary Materials**

***Supplementary Table S1.*** *Rigid body data. Range of values across the sitting still condition (run 1), for each of the six degrees of freedom.*

|  | **Participant 1** | **Participant 2** |
| --- | --- | --- |
| Right-Left | 1.9 cm | 2.4 cm |
| Back-Forward | 1.4 cm | 0.6 cm |
| Down-Up | 4.3 cm | 0.8 cm |
|  |  |  |
| Pitch | 4.3° | 2.4° |
| Yaw | 2.6° | 3.4° |
| Roll | 2.2° | 3.7° |

***Supplementary Table S2.*** *Rigid body data. Range of values across the standing still condition (run 2), for each of the six degrees of freedom.*

|  | **Participant 1** | **Participant 2** |
| --- | --- | --- |
| Right-Left | 2.5 cm | 6.5 cm |
| Back-Forward | 4.4 cm | 2.8 cm |
| Down-Up | 6.7 cm | 2.7 cm |
|  |  |  |
| Pitch | 3.7° | 12.5° |
| Yaw | 4.4° | 5.9° |
| Roll | 3.5° | 0.4° |


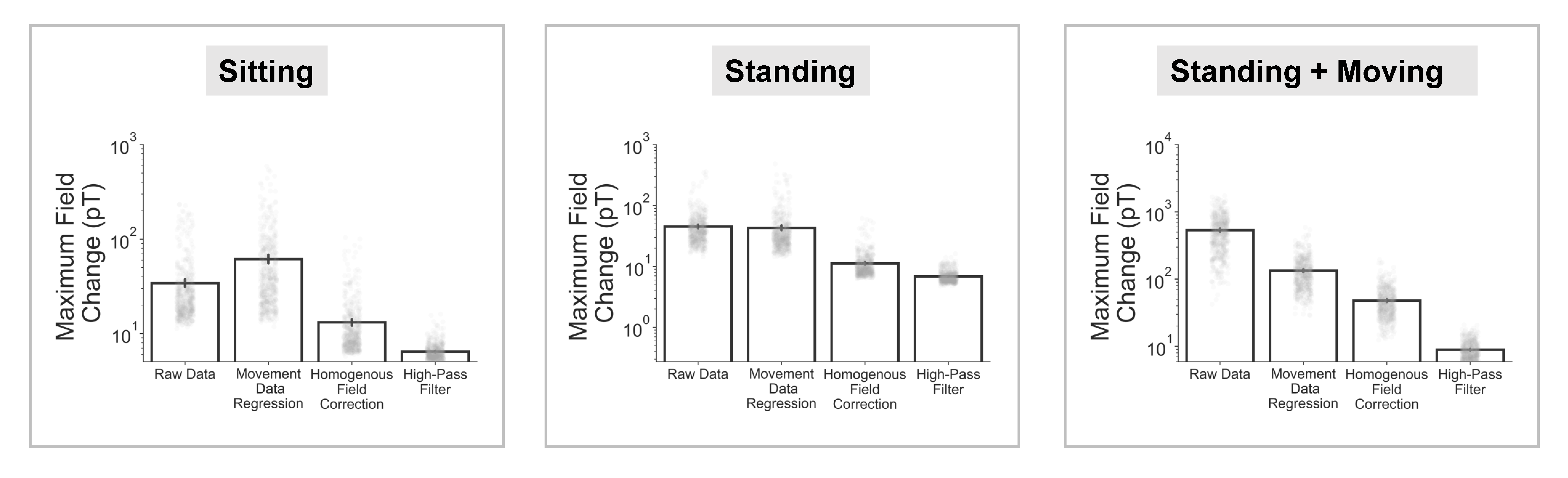


***Supplementary Fig. S1.*** *For each run the maximum change in magnetic field was calculated for each trial, using the raw data and after each of the pre-processing steps (data are averaged across the two participants). Individual data points (corresponding to each trial) are plotted in grey.*


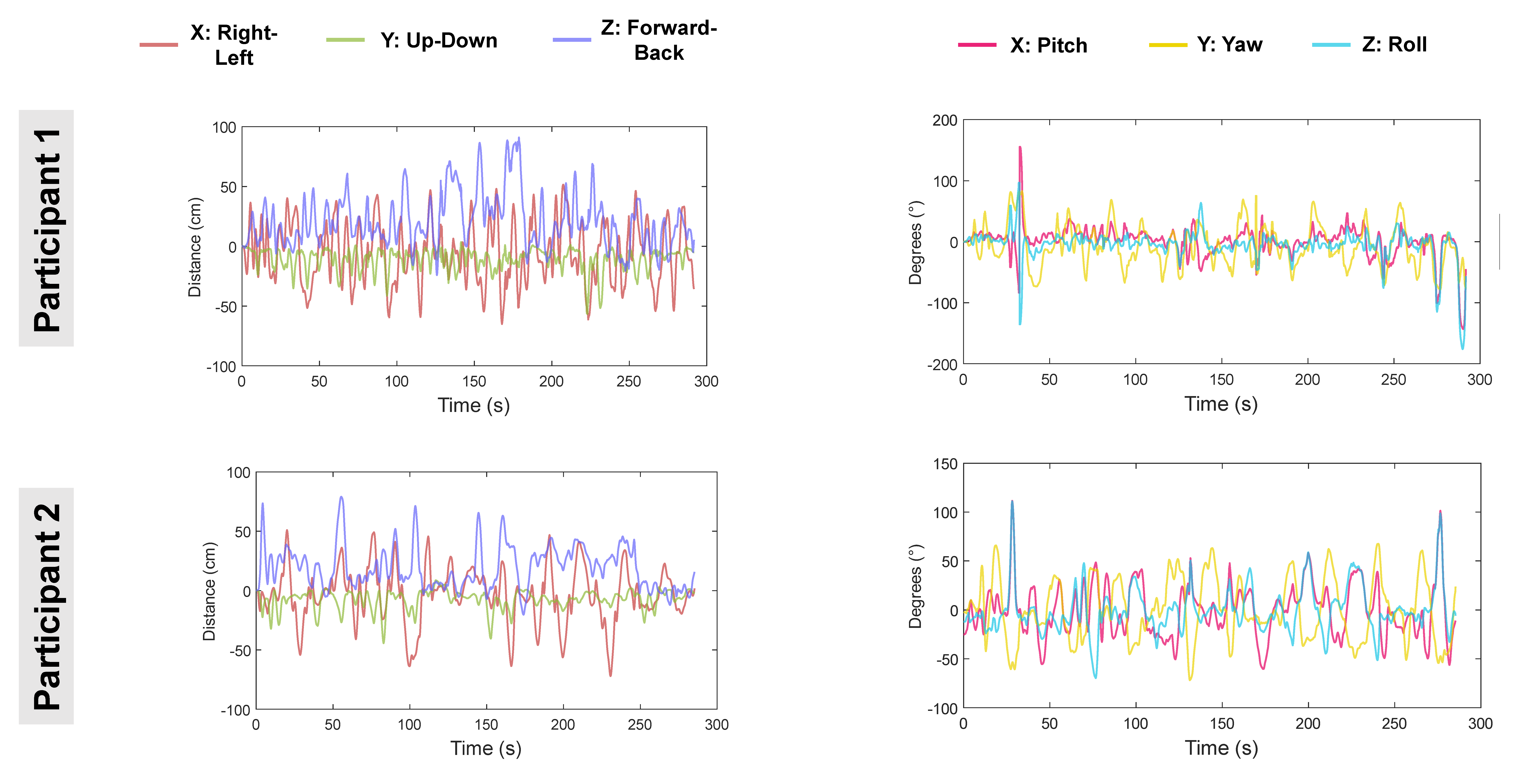


***Supplementary Fig. S2.*** *For run 3 (standing and moving), continuous rigid body data were plotted over time for both participants. Left panels = translations, right panel = rotations. Note the continuous nature of the movements over the course of the entire auditory experiment.*


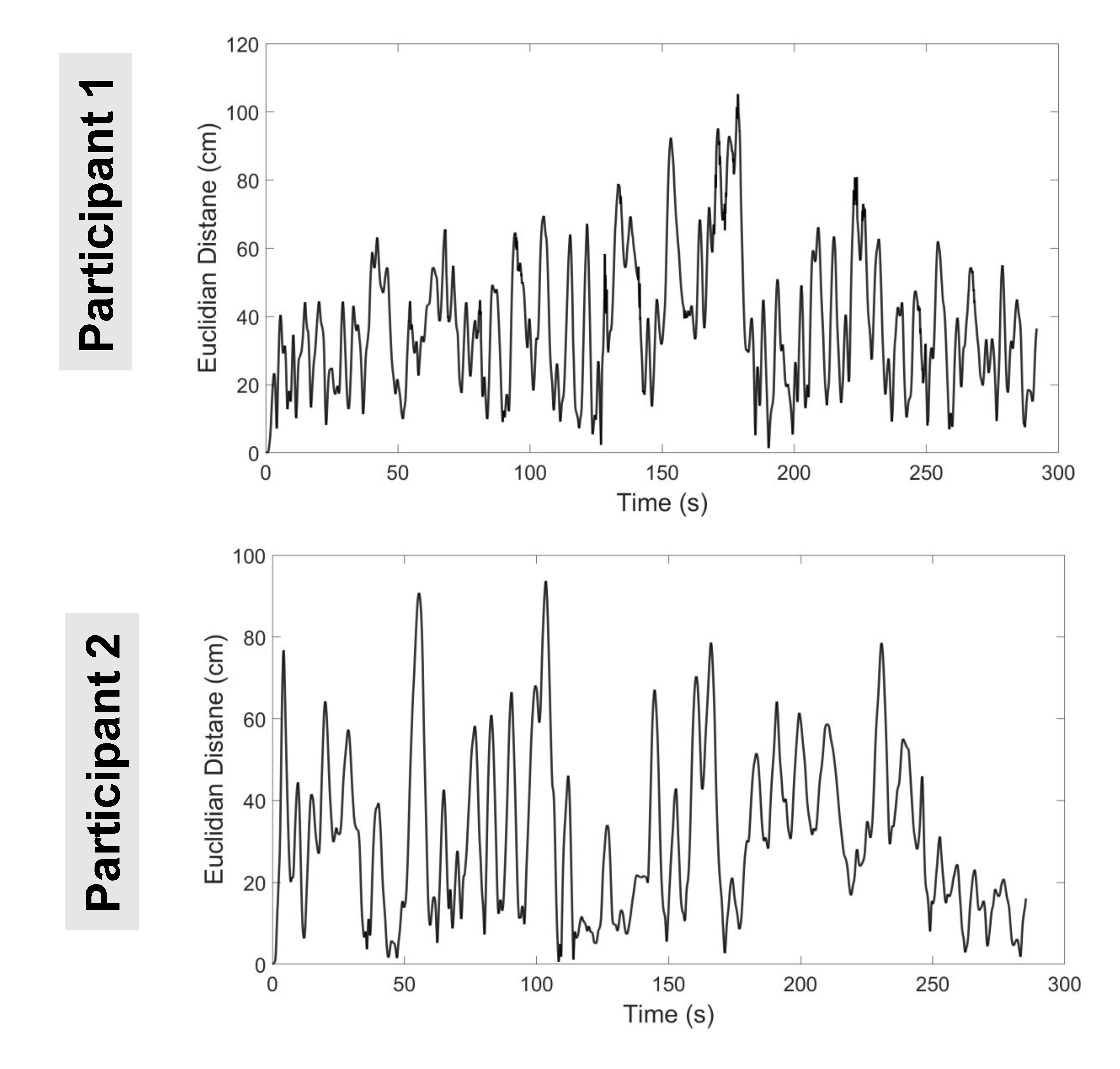


***Supplementary Fig. S3.*** *Continuous rigid body Euclidian distances from the start point of run 3, were plotted for both participants. Note the continuous nature of the movements over the course of the entire auditory experiment.*


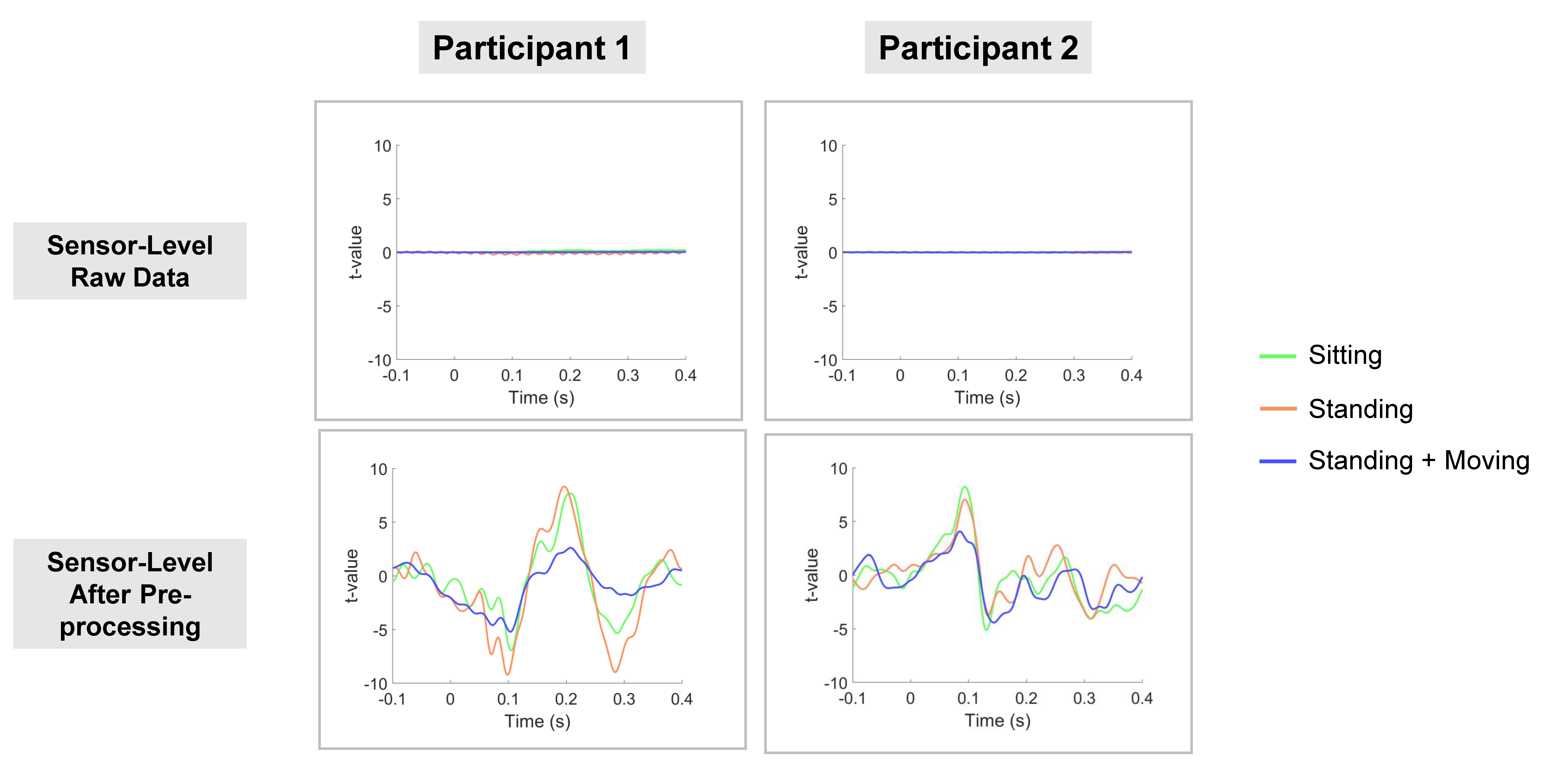


***Supplementary Fig. S4.*** *For each participant, auditory ERF t-values were calculated for the OPM sensor with the greatest M100 response using the raw data and after pre-processing. The OPM sensor was the same across all three runs (Participant 1: N3-TAN; Participant 2: 1A-TAN) and was located approximately over the left superior temporal lobe. The pipeline for pre-processing was almost identical across three runs, except that the movement data regression step was applied to only the standing and moving (run 3) data. The raw data showed no clear ERF for any run. After pre-processing (bottom panel), auditory ERFs were very similar for the sitting (green line) and standing (orange) runs, but reduced for the standing and moving run (blue line).*


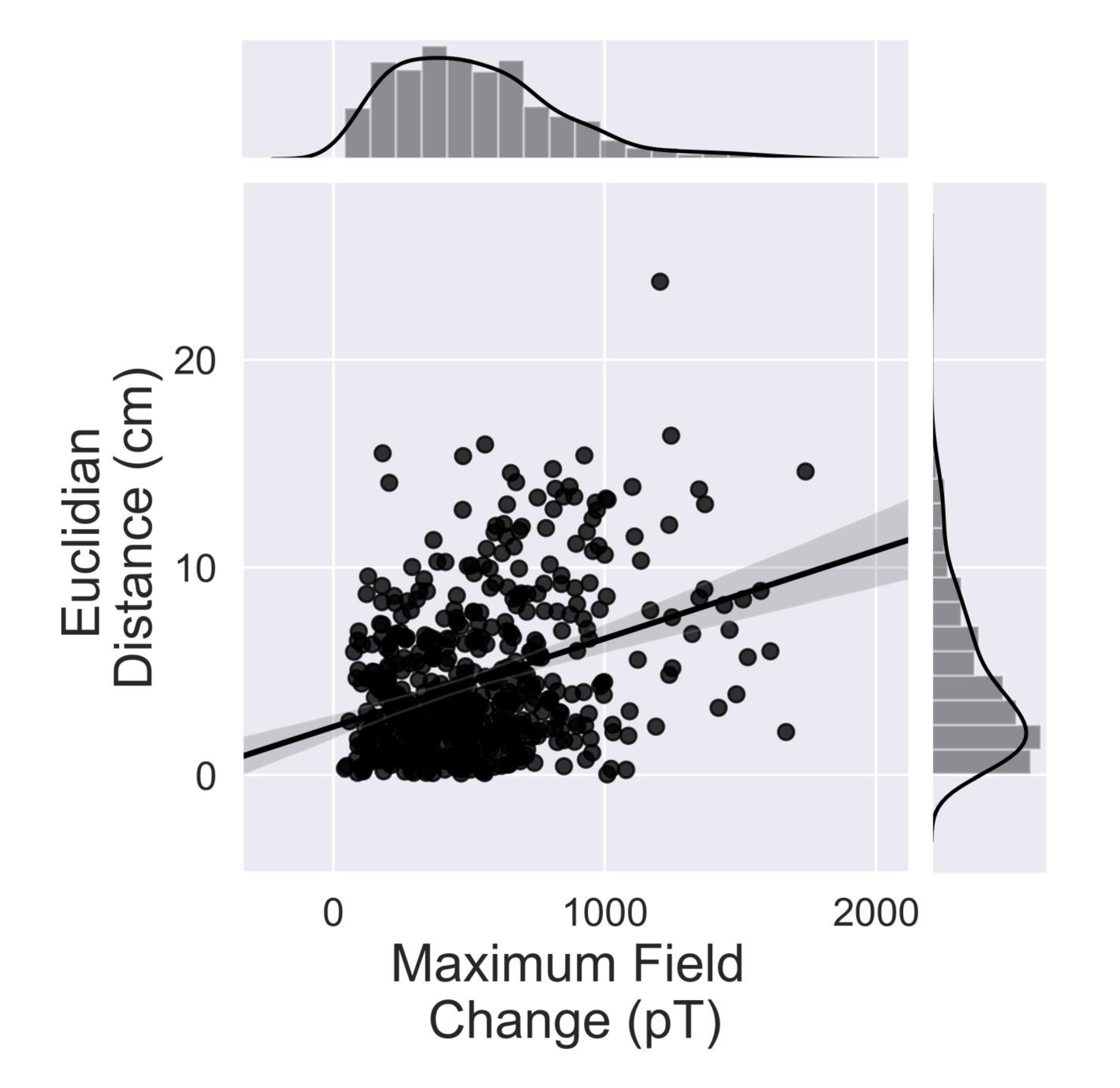


***Supplementary Fig. S5.*** *For Participant 2, a scatter-plot with regression line was produced to show the relationship between Euclidian distance moved per trial and the maximum field change. Using a Pearson’s correlation, we found no statistically significant relationship between the two, r=0.052, p=0.237.*

***
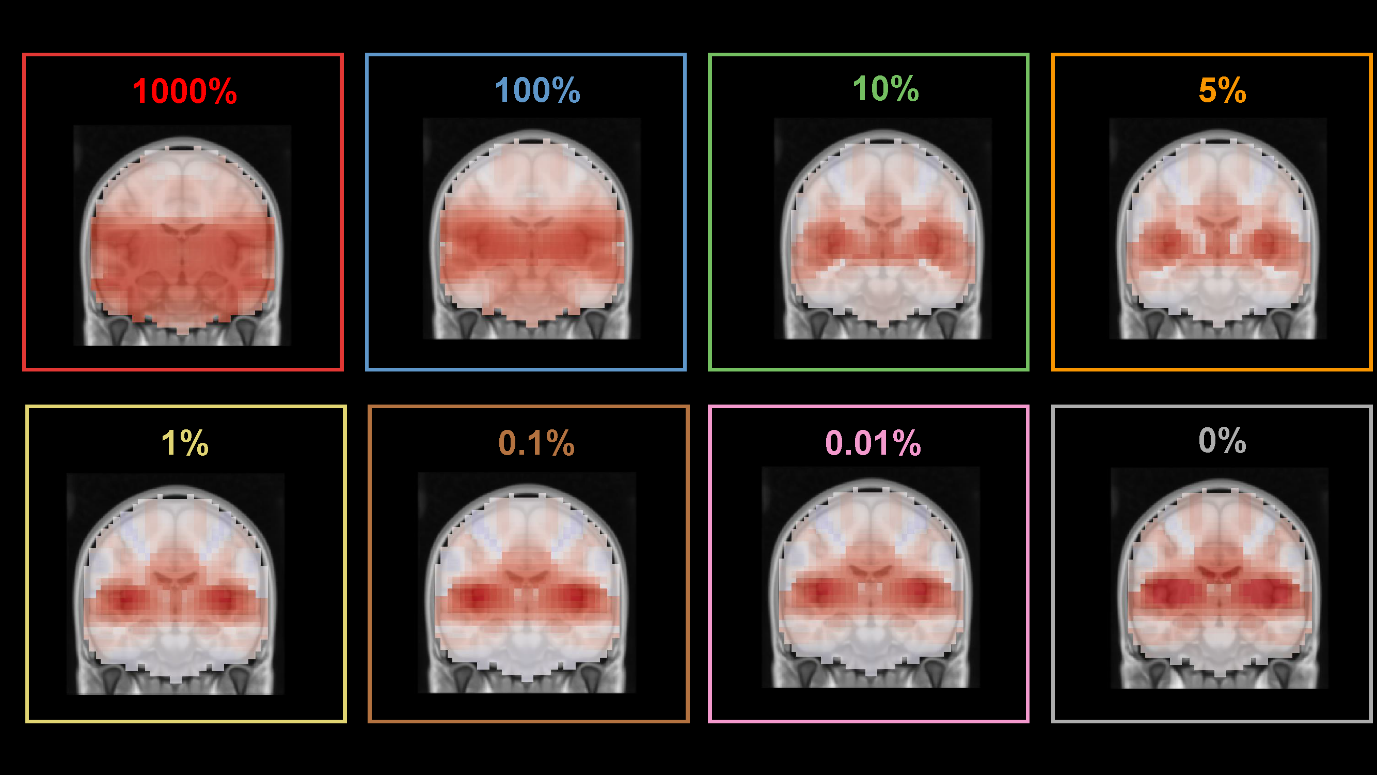
***

***Supplementary Fig. S6.***  *For participant 2, run 3 (standing and moving), whole-brain source localisation was repeated. The LCMV beamformer regularisation parameter was gradually increased from 0% to 1000%. Note the less focal whole-brain Neural Activity Index maps at 1000% and 100%.*


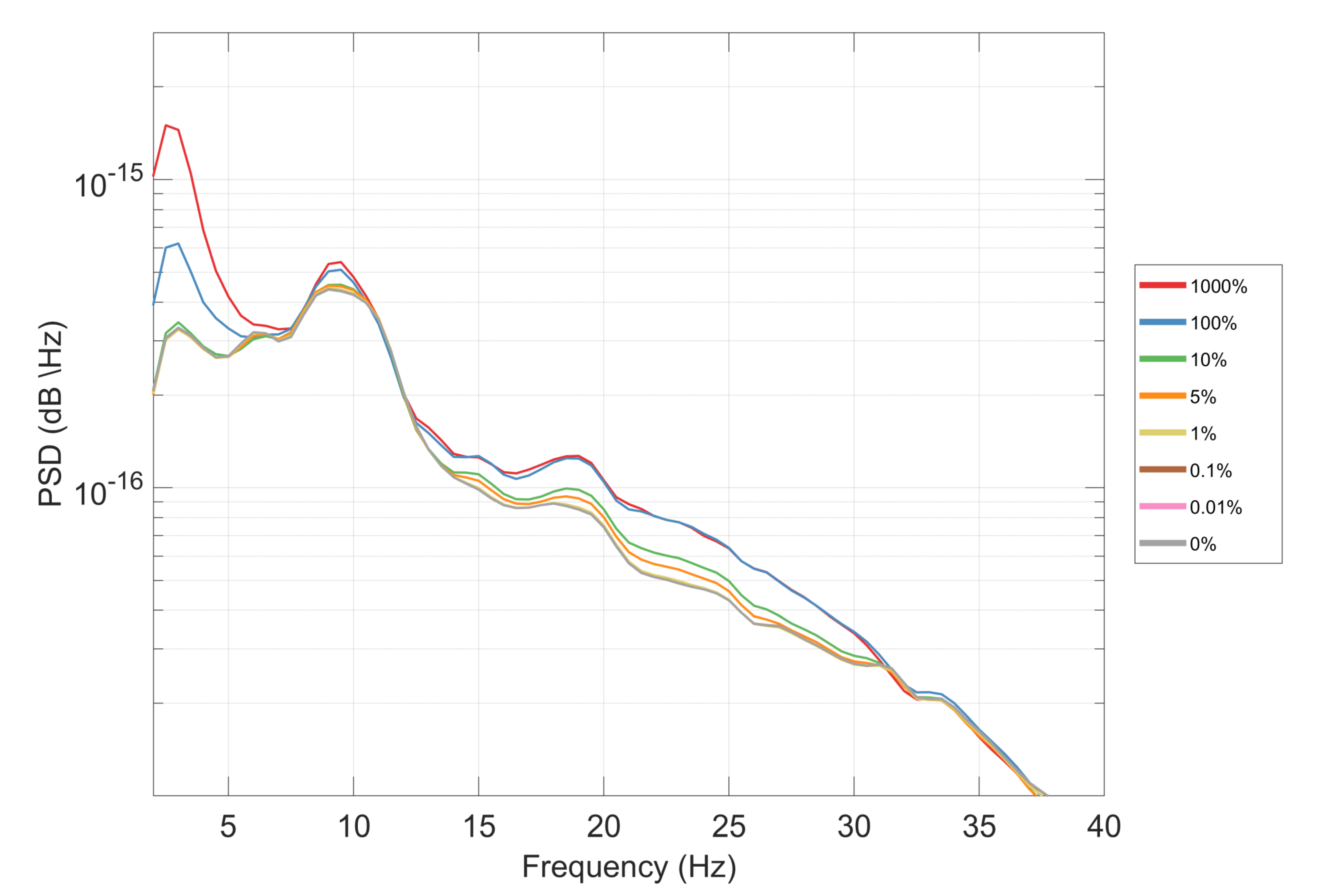


***Supplementary Fig. S7.***  *For participant 2, power spectral density (PSD) was calculated using Welch’s method using data from the auditory cortex region of interest. The LCMV beamformer regularisation parameter was gradually varied between 0% and 1000%. Note the gradual reduction in low-frequency interference suppression as regularisation increased (especially between 2-6 Hz). Also note that the lines for 0%, 0.01% and 0.1% are virtually indistinguishable on the graph, showing how similar the PSD values were between these three levels of regularisation.*


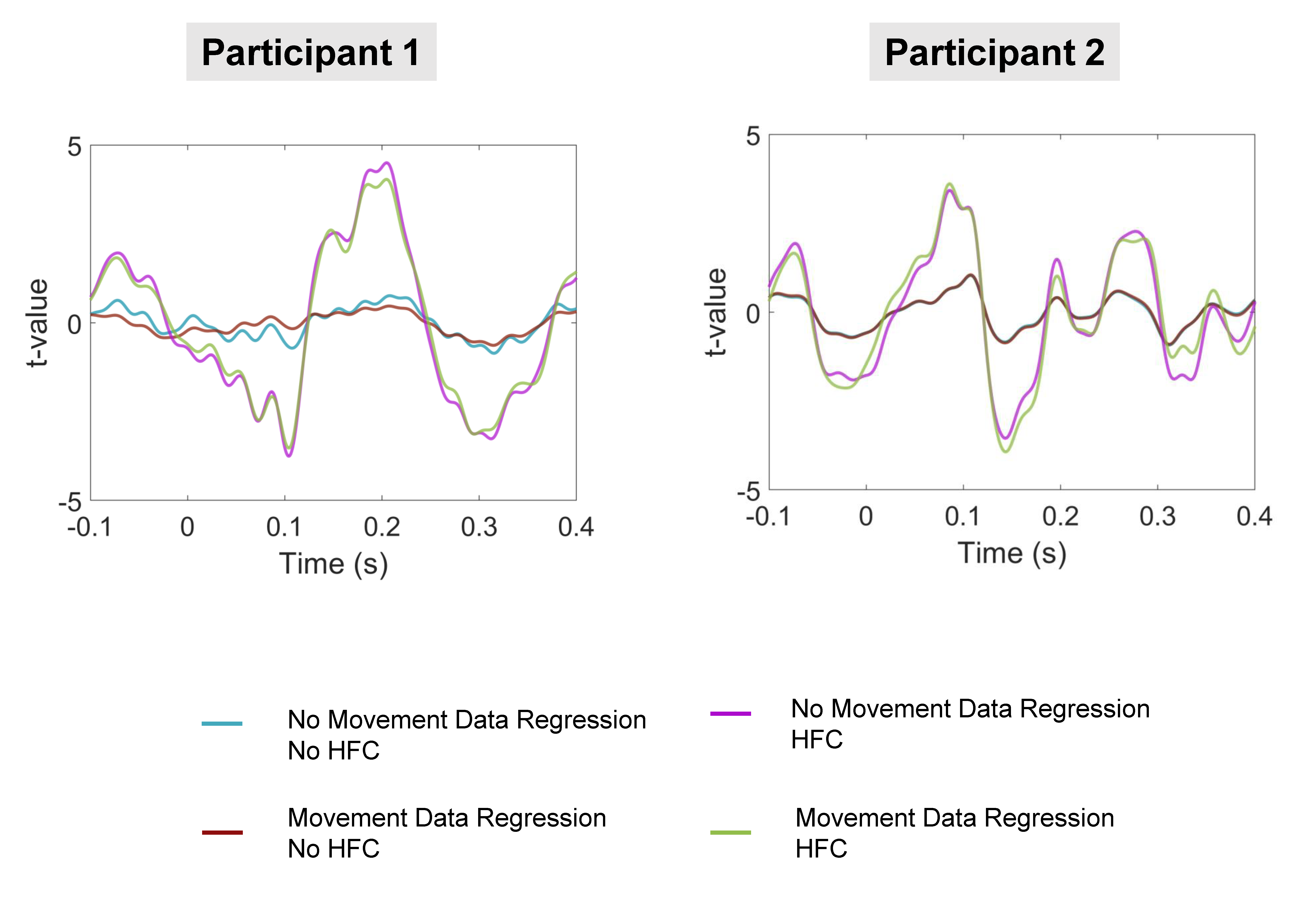


***Supplementary Fig. S8.***  *For each participant, auditory ERF t-values were calculated for the OPM sensor with the greatest M100 response using data from run 3 (standing and moving), located approximately over the left superior temporal lobe. The different coloured lines correspond to various permutations of pre-processing: with or without movement data regression, and with or without homogenous-field correction.*


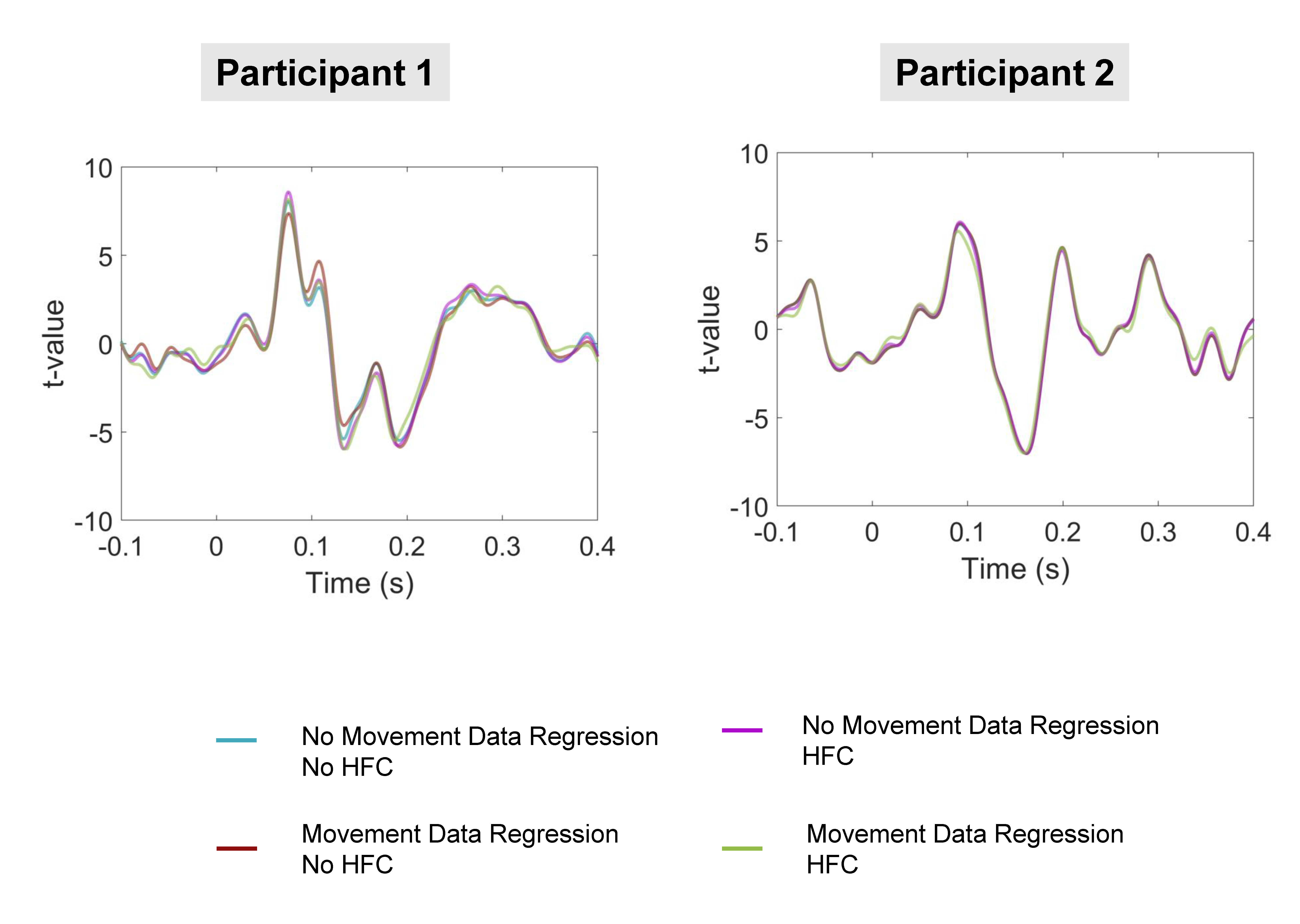


***Supplementary Fig. S9.***  *For each participant, t-values were calculated for the auditory cortex region of interest, using data from run 3 (standing and moving). The different coloured lines correspond to various permutations of pre-processing: with or without movement data regression, and with or without homogenous-field correction. Note that the lines are largely overlapping.*
