# Supplementary figures and images for: Using OPMs to measure neural activity in standing, mobile participants
Source: Neuroimage. 2021 Dec 1;244:118604. doi: 10.1016/j.neuroimage.2021.118604 (PMC8591613; doi:10.1016/j.neuroimage.2021.118604)

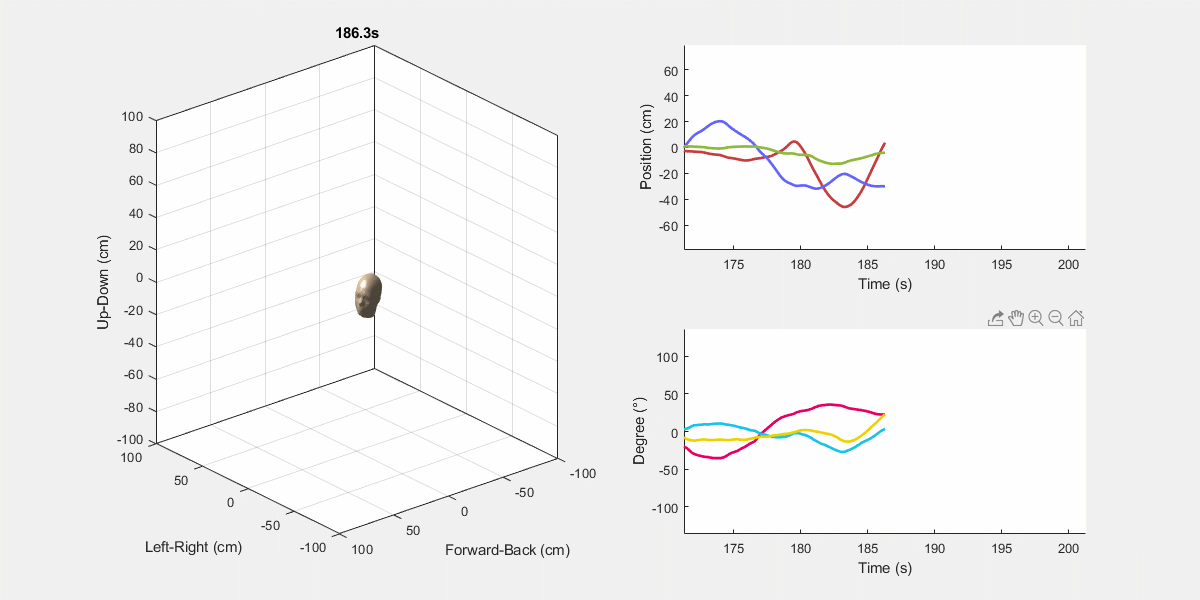

Supplement: Supplementary file 3 [file mmc3.zip › mmc3.tiff]
